# Development and validation of a GRGPI model for predicting the prognostic and treatment outcomes in head and neck squamous cell carcinoma

**DOI:** 10.3389/fonc.2022.972215

**Published:** 2023-01-12

**Authors:** Fei Han, Hong-Zhi Wang, Min-Jing Chang, Yu-Ting Hu, Li-Zhong Liang, Shuai Li, Feng Liu, Pei-Feng He, Xiao-Tang Yang, Feng Li

**Affiliations:** ^1^ Department of Head and Neck Surgery, Shanxi Province Tumor Hospital, Shanxi Hospital Affiliated to Cancer Hospital, Chinese Academy of Medical Sciences, Affiliated Tumor Hospital of Shanxi Medical University, Taiyuan, China; ^2^ Department of Anesthesiology, Shanxi Province Tumor Hospital, Shanxi Hospital Affiliated to Cancer Hospital, Chinese Academy of Medical Sciences, Affiliated Tumor Hospital of Shanxi Medical University, Taiyuan, China; ^3^ Ministry of Education, Key Laboratory of Cellular Physiology at Shanxi Medical University, Taiyuan, China; ^4^ Shanxi Key Laboratory of Big Data for Clinical Decision, Shanxi Medical University, Taiyuan, China; ^5^ Medical Data Sciences, Shanxi Medical University, Taiyuan, China; ^6^ Department of Radiology, Shanxi Province Tumor Hospital, Shanxi Hospital Affiliated to Cancer Hospital, Chinese Academy of Medical Sciences, Affiliated Tumor Hospital of Shanxi Medical University, Taiyuan, China; ^7^ Department of Cell biology, Shanxi Province Tumor Hospital, Shanxi Hospital Affiliated to Cancer Hospital, Chinese Academy of Medical Sciences, Affiliated Tumor Hospital of Shanxi Medical University, Taiyuan, China

**Keywords:** prediction, glycolysis prognosis model, head and neck squamos cell carcinoma, immune microenviroment, chemothearapeutic responses

## Abstract

**Background:**

Head and neck squamous cell carcinoma (HNSCC) is among the most lethal and most prevalent malignant tumors. Glycolysis affects tumor growth, invasion, chemotherapy resistance, and the tumor microenvironment. Therefore, we aimed at identifying a glycolysis-related prognostic model for HNSCC and to analyze its relationship with tumor immune cell infiltrations.

**Methods:**

The mRNA and clinical data were obtained from The Cancer Genome Atlas (TCGA), while glycolysis-related genes were obtained from the Molecular Signature Database (MSigDB). Bioinformatics analysis included Univariate cox and least absolute shrinkage and selection operator (LASSO) analyses to select optimal prognosis-related genes for constructing glycolysis-related gene prognostic index(GRGPI), as well as a nomogram for overall survival (OS) evaluation. GRGPI was validated using the Gene Expression Omnibus (GEO) database. A predictive nomogram was established based on the stepwise multivariate regression model. The immune status of GRGPI-defined subgroups was analyzed, and high and low immune groups were characterized. Prognostic effects of immune checkpoint inhibitor (ICI) treatment and chemotherapy were investigated by Tumor Immune Dysfunction and Exclusion (TIDE) scores and half inhibitory concentration (IC50) value. Reverse transcription-quantitative PCR (RT-qPCR) was utilized to validate the model by analyzing the mRNA expression levels of the prognostic glycolysis-related genes in HNSCC tissues and adjacent non-tumorous tissues.

**Results:**

Five glycolysis-related genes were used to construct GRGPI. The GRGPI and the nomogram model exhibited robust validity in prognostic prediction. Clinical correlation analysis revealed positive correlations between the risk score used to construct the GRGPI model and the clinical stage. Immune checkpoint analysis revealed that the risk model was associated with immune checkpoint-related biomarkers. Immune microenvironment and immune status analysis exhibited a strong correlation between risk score and infiltrating immune cells. Gene set enrichment analysis (GSEA) pathway enrichment analysis showed typical immune pathways. Furthermore, the GRGPIdel showed excellent predictive performance in ICI treatment and drug sensitivity analysis. RT-qPCR showed that compared with adjacent non-tumorous tissues, the expressions of five genes were significantly up-regulated in HNSCC tissues.

**Conclusion:**

The model we constructed can not only be used as an important indicator for predicting the prognosis of patients but also had an important guiding role for clinical treatment.

## 1 Introduction

HNSCC is a heterogeneous epithelial tumor that includes nasopharyngeal, oropharyngeal, hypopharyngeal, and laryngeal cancers. The risk factors for HNSCC include long-term alcohol exposure, smoking, betel nut chewing, chronic oral trauma, and HPV infections (1). The complexity of its etiology is a major contributor to HNSCC heterogeneity. Surgical, radiotherapy-chemotherapy, targeted therapy and immunotherapy approaches have been developed to treat HNSCC patients. However, HNSCC is associated with poor prognostic outcomes, and its 5-year OS rate is 50% (2). Therefore, there is a need to establish viable markers for the clinical prophetic prediction of HNSCC.

Recent studies have evaluated metabolic changes in tumor cells. The Warburg effect, the most prevalent and widely studied metabolic change in cancer cells, explains that under aerobic conditions, tumor tissues metabolize approximately tenfold more glucose to lactate in a given time than normal tissues, enhanced glucose uptake by tumor cells, and inhibited glucose oxidation in adjacent tissues (3). During glycolysis, glucose is converted to lactate, and cancer cells gain maximum energy. Molecular imaging revealed markedly increased glycolysis levels in HNSCC (4–6), a metabolic phenotype typical of aggressive tumor growth. This metabolic change increases glucose uptake and lactate production, affecting cell growth, proliferation, angiogenesis, and invasion (7). Overall, the oncogenic regulation of glycolysis emphasizes the biological significance of tumor glycolysis in HNSCC patients, demonstrating that targeting glycolysis remains potentially effective for clinical relevance and therapeutic intervention (8, 9). In addition, researchers have suggested that glycolysis in HNSCC is associated with alterations in oncogenes and tumor suppressor genes (10). Akt, the serine/threonine kinase, an oncogene that boosts cancer growth (11), has been proven to activate aerobic glycolysis significantly, leading to cancer cells dependent on glycolysis for survival (12). Notably, screening and identification of biological markers predicting prognosis in HNSCC by using broad glycolysis-related gene expression profiles have enormous potentially clinical relevance in targeting glycolysis for cancer therapy.

Premalignant cells frequently metastasize but are spontaneously eliminated by the immune system before developing aggressive tumors, thereby preventing tumor transformation. Thus, there is an interaction between the cancerous tissue and the immune suppressive network within the tumor microenvironment (TME). Changes in peripheral blood immune cell pool and activity are also associated with tumors (13, 14). The immune system plays a key role in carcinogenesis, development, and progression of HNSCC, where immune cell infiltration is diverse and heterogeneous. The immune system is controlled by immune checkpoint pathways that typically remain self-tolerant and limit collateral tissue damage during inflammation. Upregulated TIM-3 (15), OX40 (16), and IDO1 expressions in tumor-infiltrating lymphocytes suggest a rationale for the therapeutic targeting of these molecules. Targeting these checkpoints has led to breakthroughs in cancer immunotherapy. Immunotherapy, which activates the host’s natural defense system to identify and eliminate tumor cells, has emerged as a practical therapeutic approach. We analyzed tumor-infiltrating immune cells, immune checkpoints, and immune pathways. Our findings have clinical implications for developing personalized immunotherapeutic strategies to improve treatment outcomes for HNSCC patients.

## 2 Method and materials

### 2.1 Gene set enrichment analysis

GSEA was performed using the GSEA software (version 4.2.3) (https://www.gsea-msigdb.org/gsea/downloads.jsp) with the MSigDB glycolysis-related pathway gene sets, which contain 1320 gene sets. Pathways with p < 0.05 and FDR < 0.05 were considered significantly enriched.

### 2.2 Data collection and acquisition of glycolysis-related genes

The HNSCC gene expression data (RNA-Seq) and the corresponding clinical data (including age, gender, stage, grade, smoking, alcohol, HPV, survival time, and survival status) were downloaded from the TCGA database (https://portal.gdc.cancer.gov) and GEO dataset (https://www.ncbi.nlm.nih.gov/geo/). Used as a training cohort, the inclusion criteria for TCGA-HNSCC were: HNSCC samples with complete somatic mutation data and clinical information (457 retrieved HNSCC samples with single nucleotide polymorphism(SNP) data were analyzed), with 462 HNSCC samples and 32 adjacent non-tumor tissue samples included. The glycolysis-related gene dataset was downloaded from MSigDB. Expression characteristics of glycolysis-related genes were obtained from the MSigDB (https://www.gsea-msigdb.org/gsea/msigdb/index.jsp).

### 2.3 Identification of differential glycolysis-related genes

Using |log FC| > 1 and p < 0.05 as thresholds, differentially expressed genes between HNSCC samples and adjacent non-tumor tissue samples were evaluated using the Wilcoxon test in the limma package. Then, differentially expressed glycolysis-related genes were selected from all differentially expressed genes (DEGs) and displayed on a Venn diagram.

### 2.4 Identification and validation of a glycolysis-related gene signature

Survival-associated differentially expressed glycolytic genes were identified *via* univariate Cox regression and Lasso regression analyses, after which a polygenic prognostic risk model was constructed. Based on the median risk score of the TCGA training set as the cutoff, HNSCC patients were assigned into high- and low-risk groups. Clustering effects of Principal Component Analysis (PCA) dimensionality reduction revealed significant differences between the groups. Kaplan-Meier survival curves, time-dependent receiver operating characteristic (ROC) curves, and risk score distributions for OS prediction were evaluated to verify the prognostic significance of risk scores. Similar to the training set approach, the GEO cohort was used as an independent validation set to assess the generality and reliability of the prognostic risk model.

### 2.5 Construction of the nomogram

Independent prognostic factors in HNSCC patients were determined by univariate and multivariate Cox regression analysis. Both TCGA training set and GEO validation set were used to construct a nomogram for predicting the 1-year, 3-year, and 5-year survival outcomes of HNSCC patients. Consistency between actual survival rates and nomogram-predicted rates was tested *via* a calibration curve. In addition, decision curves were used to assess the reliability of risk scores and clinical stage.

### 2.6 Analysis of tumor immune microenvironment

The “Cell Type Identification by Estimating Relative Subsets of RNA Transcripts (CIBERSORT)”was used to assess immune cell infiltrations. The immune, stromal, and ESTIMATE (Estimation of STromal and Immune cells in MAlignant Tumors using Expression data) scores for each sample were calculated using the ESTIMATE algorithm. Correlations between the GRGPI score and those scores were determined by Spearman correlation analysis.

### 2.7 Assessment of tumor mutation burden

The tumor mutation data was obtained from the cBioPortal database. The tumor mutation burden (TMB) for all samples was calculated using “maftools” in R. Based on median TMB values, HNSCC samples were assigned into high TMB and low TMB groups. A total of 16360 genes involved in developing SNP in 457 samples were obtained by MusigCV (running under the linux system), and the top ten were screened using q<0.05 as the cut-off. Correlations between the prognosis for HNSCC patients with GRGPI and TMB were determined by Kaplan-Meier survival curves in R.

### 2.8 Analysis of drug sensitivity

To assess the clinical applicability of the established model, pRRophetic was used to calculate the IC50 of HNSCC chemotherapeutic drugs.

### 2.9 Statistical analysis

**Figure 1:**
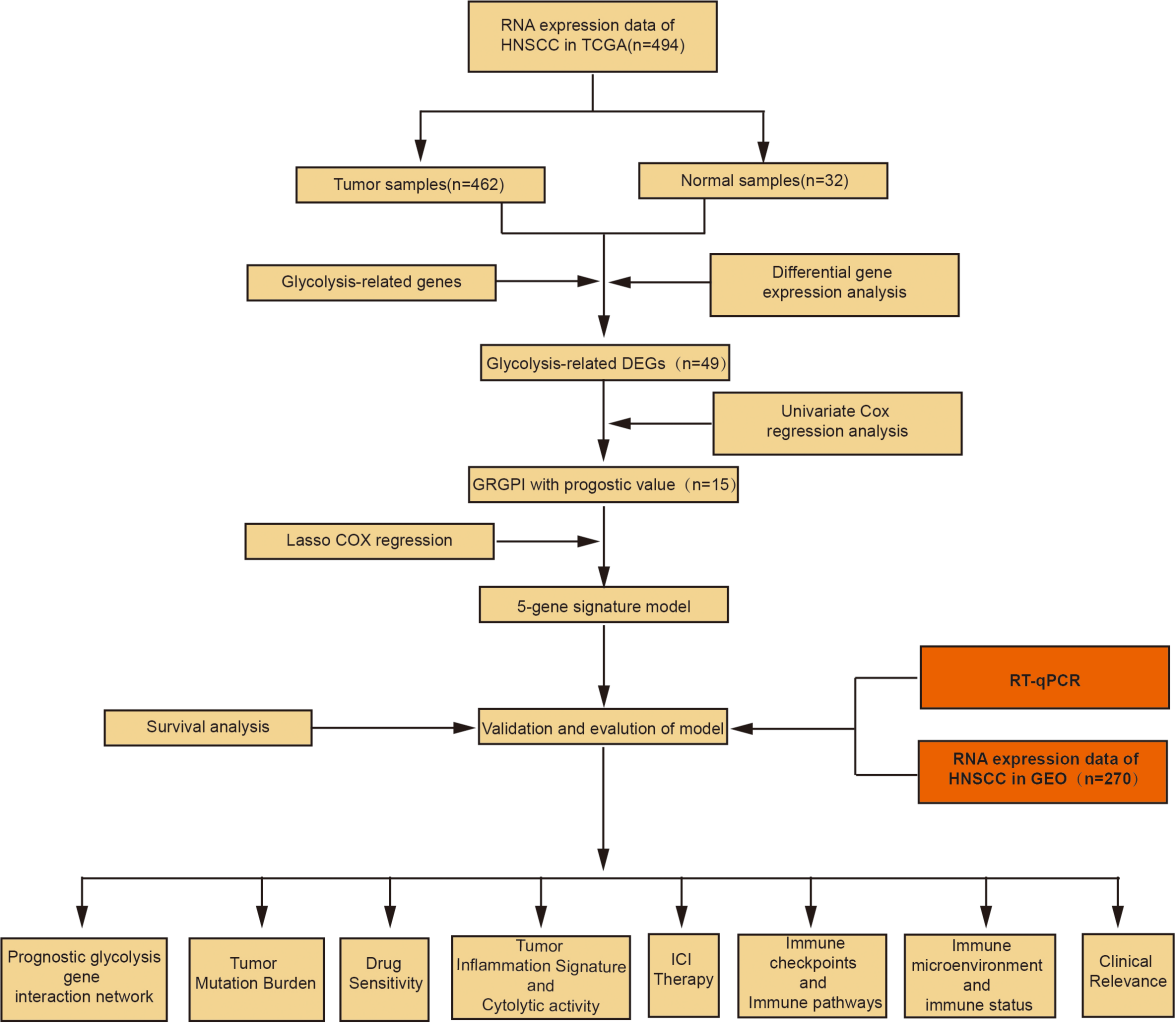
Flow chart of the study process.

The R software (version 4.1.1) was used for statistical analyses. Differentially expressed genes between tumor and adjacent normal tissues were compared by the Wilcoxon test. Survival-associated differentially expressed glycolytic genes were identified by univariate Cox and Lasso regression analyses. Then, Kaplan-Meier survival curves were plotted. Univariate COX and multivariate COX regression analyses were performed to determine the independent prognostic factors for OS. The predictive ability of the model was assessed by KM survival curves and ROC curves. Correlation tests were conducted by Spearman correlation analyses. Categorical data were compared by the chi-square test. p ≤ 0.05 was the threshold for statistical significance. The flow chart of our study is shown in **Figure 1**.

### 2.10 Reverse transcription-quantitative PCR

All HNSCC and adjacent non-tumorous tissue samples were collected from 10 patients in the Shanxi Province Cancer Hospital. Extraction of total RNA from HNSCC tissues and adjacent non-tumor tissues was performed by the TRIzol reagent (Invitrogen, CA, USA). cDNA synthesis from the extracted RNA was performed by PrimeScriptTM RT Master Mix (RR036B, Takara). We use Quantitative PCR to analyze the mRNA expression levels of the prognostic glycolysis-related genes by GoTaq^®^ qPCR Master Mix (Promega, A6001). The RT-qPCR was utilized in the ABI Vii7 Sequence detection system (ABI, USA). The PCR reaction system and conditions were according to the manufacturer’s instructions. Gene expression levels of STC1, STC2, AURKA, P4HA1, and PLOD2 were calculated using the 2-ΔΔCT method.

## 3 Results

### 3.1 GSEA

Based on KEGG, REACTOME and HALLMARK gene sets, GSEA was performed to reveal potential differences between HNSCC and control groups. These pathways are associated with glycolysis, implying that glycolysis plays an essential role in HNSCC (**Supplementary Figures 1A–C**).

### 3.2 Identification of glycolysis-related DEGs

**Figure 2:**
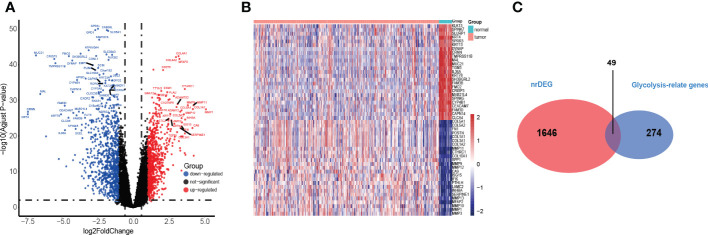
Identification of the HNSC-related DEGS in TCGA. **(A, B)** The volcano and heatmap plot showed differentially expressed genes between tumor and adjacent normal tissue. **(C)** Venn diagram showed glycolysis-relate differentially expressed genes between tumor and adjacent normal tissue.

A total of 1695 differentially expressed genes (DEGs) (149 upregulated and 119 downregulated genes) in the TCGA training cohort were identified by Wilcoxon signed-rank test and visualized using volcano plots (**Figure 2A**) and heatmaps (**Figure 2B**). Then, 49 glycolysis-related genes were extracted from the DEGs (**Figure 2C**).

### 3.3 Construction of glycolysis-related gene signature for predicting patient outcomes

**Figure 3:**
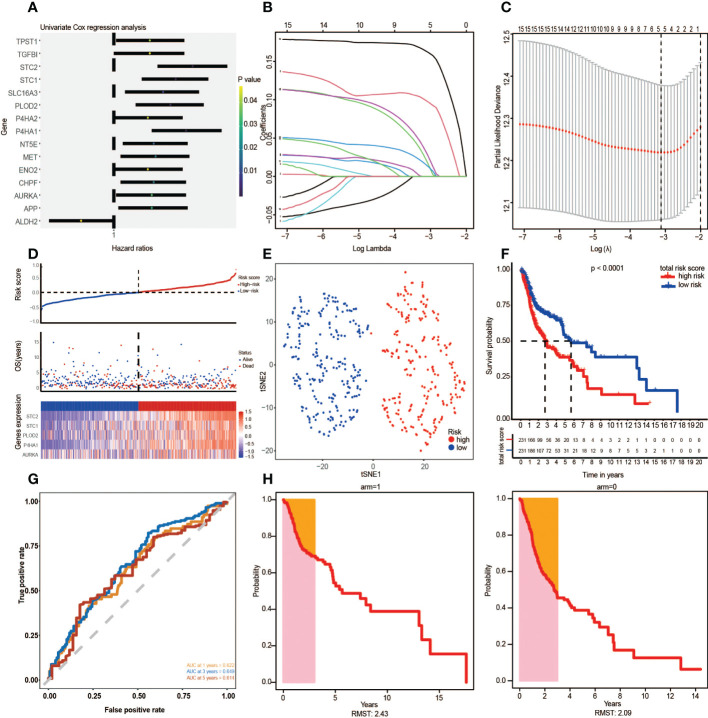
The Glycolysis-Related Gene Signature on the training cohort was constructed to predict patient outcomes. **(A)** Univariate Cox regression analysis yielded 15 prognosis-associated differentially expressed glycolysis-related genes. **(B, C)** LASSO regression analysis identified the five prognostic genes. **(D)** The TCGA risk score, survival time, survival status, and expression of the five-gene signature. **(E)** t-SNE cluster showed groups with high and low-risk scores. **(F)** Kaplan-Meier survival curve analysis for HNSCC patients divided into high-risk and low-risk groups. **(G)** Time-independent ROC curve of a risk score for prediction of 1-year, 3-year, and 5-year overall survival outcomes. **(H)** RMST plot for the TCGA training set.

Through univariate Cox regression analysis, 15 prognosis glycolytic genes were established to be closely associated with survival outcomes of HNSCC patients (**Figure 3A**). The 15 OS-related genes may be collinear rather than independent. LASSO Cox regression analysis was performed to determine the real OS-affecting factors, and finally, a prognostic panel consisting of five glycolysis-related genes was established. The risk score was calculated as: Riskscore=0.021*AURKA+0.099*P4HA1+0.015*PLOD2+0.031*STC1+0.163*STC2. (**Figure 3B, C**). Based on this gene signature, all patients were assigned to high (n=231) and low-risk (n=231) subgroups using the risk score median as the threshold. Risk scores, survival scores, and heatmap of prognostic glycolytic gene expressions among the low-risk and high-risk patients are presented in **Figure 3D**. Based on expressions of these five hub genes, dimensionality reduction was performed in all patients and presented with methods of t-distributed stochastic neighbor embedding (t-SNE), suggesting that different risk subgroups show significant discrete tendencies directly in the two-dimensional plane (**Figure 3E**). Kaplan-Meier survival curves revealed that high-risk score patients had significantly worse OS outcomes than low-risk score patients. The area under the curve (AUC) analysis for HNSCC patients at 1-year, 3-year, and 5-year revealed respective OS rates of 0.622, 0.649, and 0.614, demonstrating the optimal predictive performance of GRGPI (**Figures 3F, G**). Finally, the year with the largest AUC value is shown in the RMST plot (**Figure 3H**).

### 3.4 Verification of the five gene signature using the validation cohort

**Figure 4:**
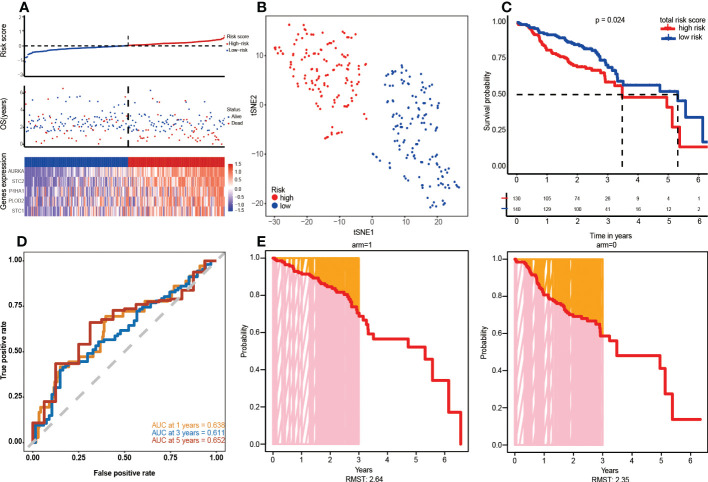
Verification of the five-gene signature in the validation cohort (GSE65858). **(A)** Risk map of patients based on risk score heatmap of survival status and risk gene expression profiles of individual HNSCC patients. **(B)** t-SNE grouping cluster. **(C)** Kaplan–Meier curves according to the five-gene signature. Log-rank tests were performed to determine the p values. **(D)** ROC curve and AUC values of five-gene feature classification in GEO. **(E)** RMST plot for the GEO testing set.

Given that the predictive potential of GRGPI in different datasets is misty into account, GSE65858 was used as the independent validation set. Based on the above risk scores, patients were assigned to low-risk (n=140) and high-risk (n=130) groups (**Figure 4A**). Findings from t-SNE and KM survival analyses of the GEO validation set were consistent with the results of the TCGA training cohort (**Figures 4B, C**). The AUC values for ROC curves accurately revealed the predictive performance of the prognostic risk model, with the largest AUC value year shown as an RMST plot (**Figures 4D, E**).

### 3.5 Independent prognostic, predictive value of risk scores and construction of the nomogram

**Figure 5:**
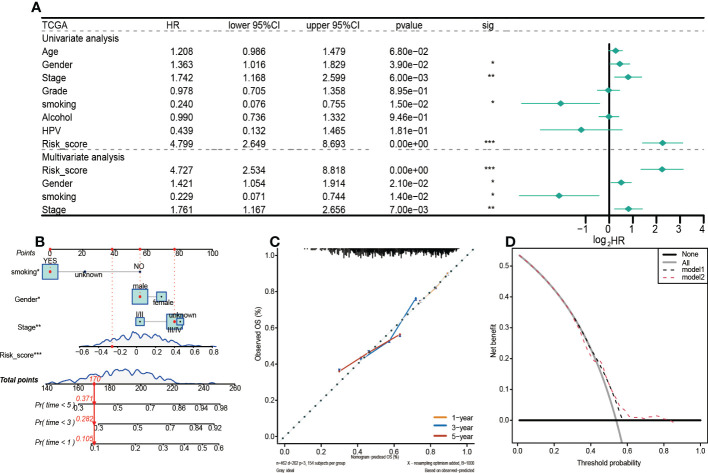
Prognostic values of the 5-gene signature model in the TCGA training set. **(A)** Results of univariate and multivariate Cox regression analyses regarding OS. **(B)** Nomogram for prediction of 1-year, 3-year, and 5-year survival probabilities of HNSCC patients. **(C)** Calibration curve for assessing the agreement between nomogram predicted and actual survival outcomes. **(D)** Assessment of the reliability of risk scores by DCA (decision curve). (*p < 0.05, **p < 0.01, ***p < 0.001).

In this study, the risk score, gender, smoking, and clinical stage were established to be independent prognostic factors in HNSCC patients, and they were used to construct subsequent nomograms (**Figure 5A**). Nomograms were used to predict the 1-year, 3-year, and 5-year survival probabilities of HNSCC patients (**Figure 5B**). Moreover, a calibration curve was constructed to assess the agreement between nomogram predictions and actual survival outcomes (**Figure 5C**). The actual and predicted survival rates at 1-year, 3-year, and 5-year were well matched, indicating that the nomogram has a good predictive performance. A decision curve (DCA) was used to assess the reliability of the risk score. It was observed that Model1 (Stage) was close to the extreme curve, while Model2 (RiskScore) was significantly higher than the extreme curve (**Figure 5D**).

**Figure 6:**
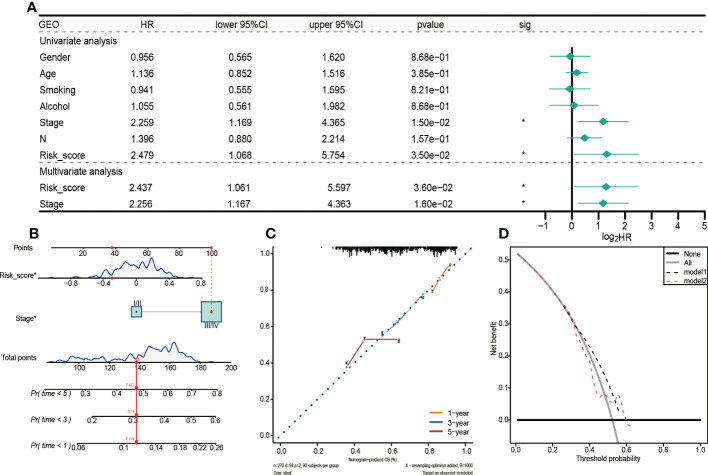
Prognostic values of the 5-gene signature model in the GEO set. **(A)**Univariate and multivariate Cox regression analysis to investigate the independence of risk models among clinicopathological factors. **(B)** Nomogram for predicting the 1-year, 3-year, and 5-year survival probabilities of HNSCC patients. **(C)** Calibration curve for assessing the agreement between nomogram predicted and actual survival outcomes. **(D)** Decision curve analyses of the nomogram based on OS outcomes.

The above analyses were also performed on the GEO validation set to verify the robustness of the model (**Figure 6A**). Unlike the TCGA training set, univariate and multivariate Cox regression analyses revealed that in the GEO validation set, only risk score and clinical stage were independent prognostic factors for HNSCC patients. Therefore, a nomogram integrating risk scores and clinical stages was constructed to predict the 1-year, 3-year, and 5-year survival probabilities of HNSCC patients (**Figure 6B**). Findings from the calibration curve were consistent with those of the TCGA training set (**Figure 6C**).

### 3.6 Clinical relevance form

Based on the relationship between high and low-risk groups and clinical stages in the TCGA training set, a clinical correlation table was prepared. It was established that about 60% of patients in the low-risk group were in locations I/II, while 76% of patients in the high-risk group were in stages III/IV (**Supplementary Figure 2**), implying that risk grouping was positively correlated with the clinical stage. These findings prove that the constructed GRGPI model is clinically valuable.

### 3.7 The tumor mutation burden

**Figure 7:**
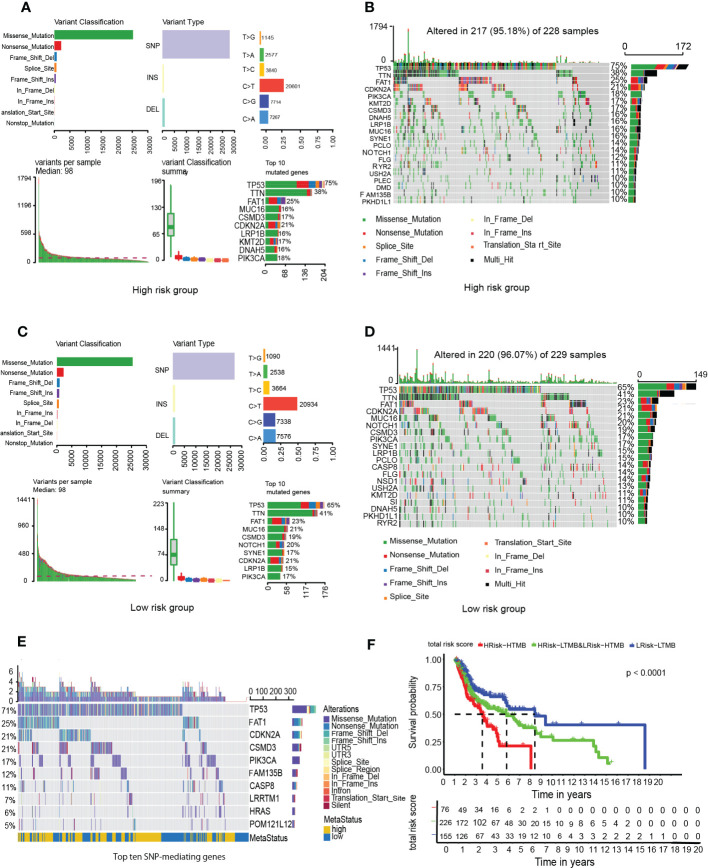
Analysis of tumor mutation burden among HNSCC patients. **(A)** High-GRGPI. **(B)** High-GRGPI group mutation types and top 20 mutated genes in the sample. **(C)** Low-GRGPI. **(D)** low-GRGPI group top 20 mutated genes. **(E)** Mutation types of the top ten SNP-driven genes and their distribution in high-GRGPI group and low-GRGPI group. **(F)** Kaplan-Meier survival curve showing OS differences among the three subgroups.

Mutation data for HNSCC were downloaded from the cBioPortal database. Somatic mutation types for the 457 patients were evaluated, and SNP was found to be the most dominant mutation type (**Figures 7A, C**). About 95.18% of samples in the high-risk group had SNPs, compared to 96.07% in the low-risk group (**Figures 7B, D**).

Given that SNP was the most dominant mutation type in HNSCC, 16,360 SNP-mediating genes were identified in the 457 samples using MusigCV. Using q<0.05 as the cut-off, the top ten genes were screened. Mutations types of the 10 genes and their distributions in high-risk and low-risk groups were analyzed (**Figure 7E**). The top ten genes with the highest mutation rates in the high-risk group were TP53, TTN, FAT1, MUC16, CSMD3, CDKN2A, LRP1B, KMT2D, DNAH5, and PIK3CA (**Figure 7B**), while those in the low-risk group were TP53, TTN, FAT1, MUC16, CSMD3, NOTCH1, SYNE1, CDKN2A, LRP1B, and PIK3CA (**Figure 7D**). It was observed that the gene with the highest mutation rate was TP53 in HNSCC patients regardless of the high GRGPI group or the low GRGPI group, suggesting that the mutations of the tumor suppressor gene TP53 may have potential clinical and pathophysiological significance in HNSCC patients. In fact, in a recent study, the mutational profile of TP53 has been proved to act as an independent prognostic factor in HNSCC patients. This relationship is associated with unique site-specific biological networks, consistent with our findings (17). Correlation analyses showed that GRGPI was positively correlated with TMB (R=0.015, p=0.75). The difference in the number of HNSCC patients in the high and low TMB groups was insignificant (**Supplementary Figure 3A**). Moreover, the difference in TMB values between the groups was negligible (**Supplementary Figure 3B**). We combined GRGPI and TMB and grouped them into three; high GRGPI high mutation (HTMB+HGRGPI), high GRGPI low mutation or low GRGPI high mutation (HTMB+LGRGPI & LTMB+HGRGPI), and low GRGPI low mutation (LTMB+LGRGPI). Then, Kaplan-Meier survival curves were drawn. The survival curve showed that the LTMB+LGRGPI group had the best prognosis, while the HTMB+HGRGPI group had the worst prognosis (**Figure 7F**). These findings imply that high GRGPI and high TMB play a synergistic role in promoting tumor occurrence and development, and the combined effects of the two may lead to worse prognostic outcomes.

### 3.8 Prognostic glycolysis gene interaction network

Interactions among the five glycolysis key genes and transcription factors may elucidate on mechanisms of the GRGPI model. Using cor Filter=0.5 and fdr Filter=0.01 as critical values, associations between AURKA, P4HA1, PL0D2 and 11 transcription factors were obtained (**Supplementary Figure 4**), which proved that the genes used to construct the GRGPI model were correlated with transcription factors in cancer and para cancer differentially expressed genes.

### 3.9 The immune microenvironment and immune status

**Figure 8:**
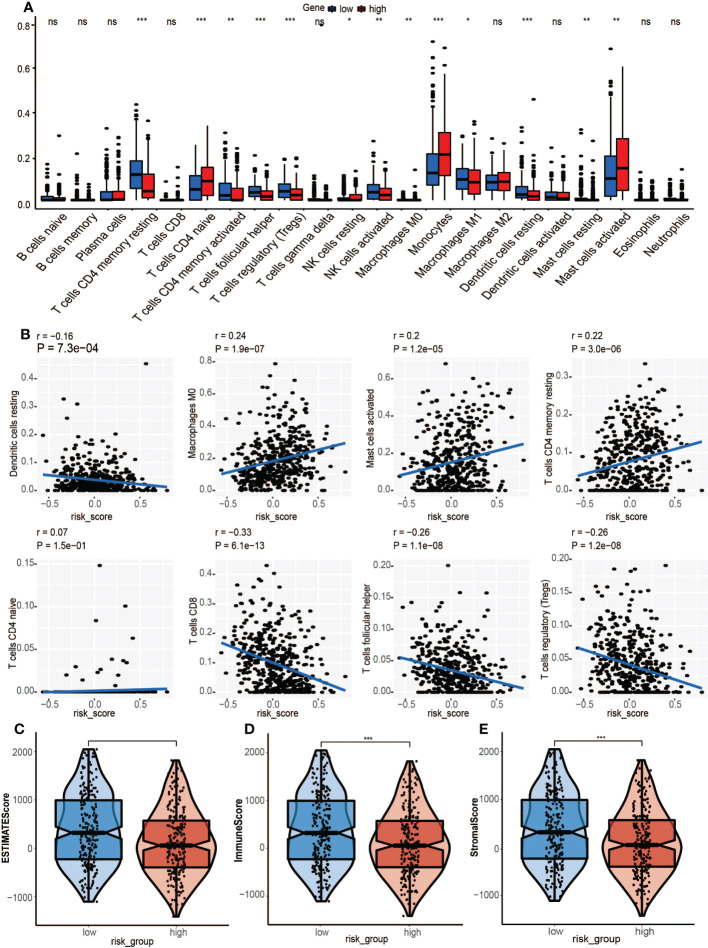
Association between tumor immunity and GRGPI scores in high and low GRGPI groups. **(A)** The 22 infiltrating immune cells are shown in boxplots. **(B)** Correlation analysis between 8 types of infiltrating immune cells and GRGPI scores. **(C–E)** Boxplots showing the correlation between GRGPI with ESTIMATE, immune, and stroma scores of HNSCC samples. (ns, not significant, *p < 0.05, **p < 0.01, ***p < 0.001).

Compared to the low-risk group, infiltrations of resting CD4 memory T cells, M0 macrophages, M2 macrophages, and activated mast cells were marked in the high-risk group, while infiltrations of CD8 T cells, follicular helper T cells, and Treg cells were to a greater extent (**Figure 8A**). Cell immunity-related cells, such as CD8 T cells, were highly infiltrated in the low-risk group, suggesting that immune cells may be activated in the low-risk group and suppressed in the high-risk group. Moreover, the M0 macrophages, activated mast cells, and resting CD4 memory cells were positively correlated with GRGPI scores while resting dendritic cells, CD8 T cells, follicular helper T cells, and Treg cells were negatively correlated with GRGPI scores (**Figure 8B**). The higher the GRGPI scores, the worse the extent of T cell infiltrations, validating that weaker antitumor immunity may be one of the reasons for poor prognostic outcomes. Therefore, the high-risk group was defined as the low-immunity group, while the low-risk group was defined as the high-immunity group. Differences in ESTIMATE scores between the high-risk group and low-risk group were insignificant. However, the high-risk group exhibited low immune scores (p< 0.001, **Figure 8D**). These findings are consistent with those obtained from CIBERSORT, whereby the high-risk group exhibited worse immune status while the low-risk group exhibited better immune status. The relationship between stromal cells and GRGPI scores was further investigated (18). The high-risk group had higher stromal scores (p < 0.01, **Figure 8E**), implying that tumor stroma plays an important role in tumor development.

### 3.10 Immune checkpoints and immune pathways

**Figure 9:**
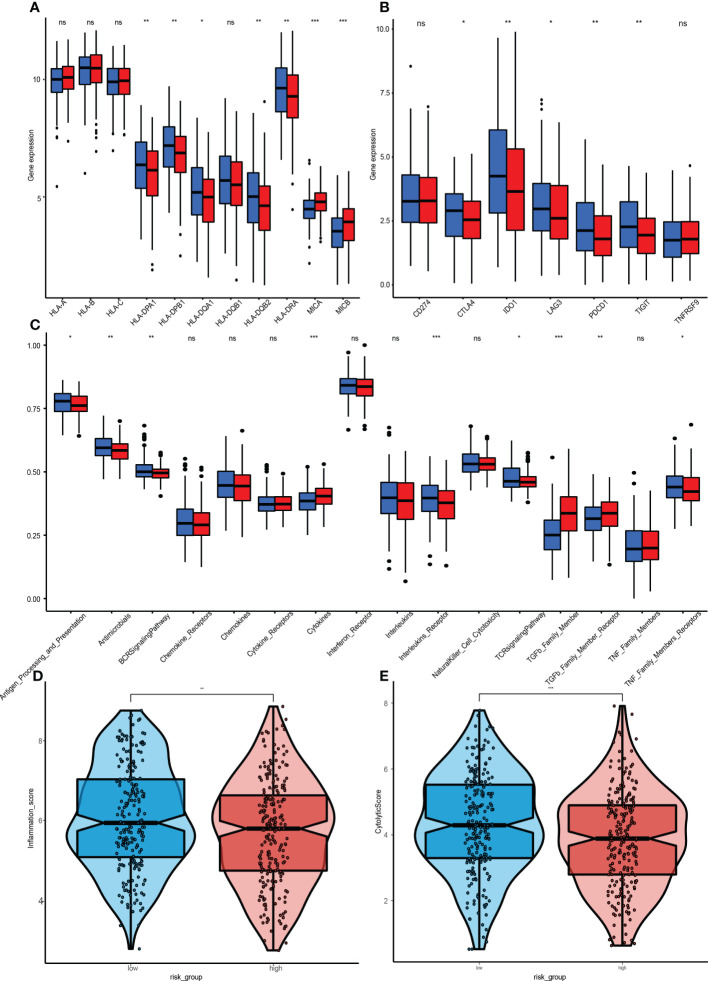
Immunization between high and low risk groups. **(A)** Differential expressions of 11 HLA class immune checkpoints. **(B)** 7 genes. CD274, CTLA4, IDO1, LAG3, PDCD1, TIGIT, and TNFRSF between the high-GRGPI group and low-GRGPI group. **(C)** Immune-related pathways. **(D, E)** Violin diagram for differences in cytolytic activities and Tumor Inflammation Signature between the high-GRGPI and low-GRGPI groups. (ns, not significant, *P<0.05, **p < 0.01, **p < 0.001).

ICI therapy has advanced the treatment of many solid tumors. Therefore, 11 human leukocyte antigen(HLA) class immune checkpoints were included, and their differential expressions in high-risk and low-risk groups were determined. Four HLA class checkpoints (HLA-A, HLA-C, MICA, and MICB) were highly expressed in the high-risk group (**Figure 9A**), while the remaining seven were highly said in the low-risk group. Since the HLA class immune checkpoints are closely associated with immune responses, the better prognostic outcomes in the low-risk group could have been due to better immune responses. The expressions of 7 genes (CD274, CTLA4, IDO1 LAG3, PDCD1, TIGIT, and TNFRSF9) in the high-risk group and low-risk group were also analyzed (**Figure 9B**). Results show that five immune checkpoints cut in the high-risk group, CTLA4, IDO1, LAG3, PDCD1, and TIGIT, which are consistent with the result, once again proved that the GRGPI model and the close correlation between HLA class immune checkpoints.

GSEA was performed to assess the immune pathways, and differentially expressed immune-related pathways between the high-risk and low-risk groups were obtained (**Figure 9C**). The BCR, Chemokines, Chemokine, Receptors, Interleukins Receptor, NK cell Cytotoxicity and TCR signalling pathway were found to be enriched in the high-risk group. However, enrichments of “TGFβ Family Members” and “TGFβ Family Members Receptor” were significantly high in the low-risk group, in accordance with the functions of TGF-β, which is involved in tumorigenesis and immunosuppression.

### 3.11 Tumor inflammation signature

The Tumor Inflammation Signature (TIS) is investigational use only (IUO) 18-gene signature that measures pre-existing but suppressed adaptive immune responses within tumors (19). The high-risk group had a low TIS score, implying that this group had weaker adaptive immune responses and worse prognostic outcomes (**Figure 9D**).

### 3.12 Cytolytic activity

The CYT score is a novel cancer immune index calculated from mRNA expressions of GZMA and PRF1 (20). The transcriptional levels of GZMA and PRF1 were determined to assess the cytolytic activities of immune lymphocytes in HNSCC. Based on previous risk grouping, the low-risk group exhibited a higher CYT score (**Figure 9E**), implying that immune cells in the low-risk group had stronger cytolytic activities and anti-tumor immune response, leading to a better prognosis.

### 3.13 GRGPI was highly predictive in ICI therapy

**Figure 10:**
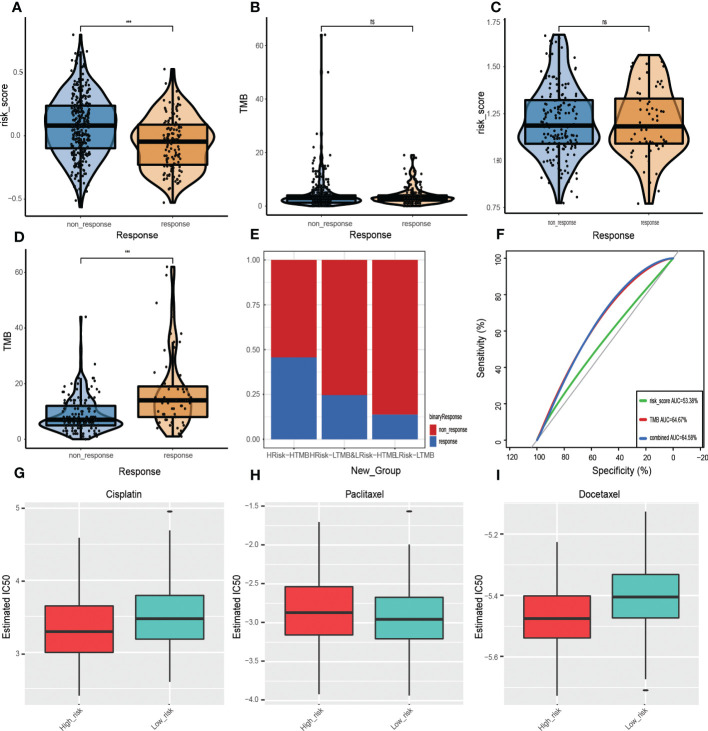
Prognostic value of ICI therapy. **(A, B)** Sizes of training cohort responses, non-response sample GRGPIs, and TMB values. **(C, D)** Differences in GRGPIs and TMB between responsive and non-responsive samples from IMvigor210. **(E)** Proportions of immunotherapy-responsive and non-responsive samples in the three subgroups from the IMvigor210 cohort. **(F)** ROC curves of GRGPI, TMB, and GRGPI combined with TMB in the IMvigor210 cohort. Analysis of drug sensitivity. Differences in IC50 values of **(G)** Gefitinib, **(H)** Erlotinib, and **(I)** Cisplatin in the high-GRGPI group and low-GRGPI group.

TIDE is a computational framework developed by Peng Jiang et al. to identify two tumor immune escape mechanisms (21). A higher TIDE score means a greater likelihood of immune evasion, indicating that a patient is less likely to benefit from ICI therapy and may have worse prognostic outcomes. The TIDE website was used to process 457 HNSCC samples with complete somatic mutation data in the training cohort, of which 131 responded to immunotherapy while the remaining 326 did not. Then, the GRGPIs of responding and non-responding samples were evaluated, which revealed that responding samples had lower GRGPIs (**Figure 10A**). This confirms our findings in a previous study. Since the low-risk group had better performance in immune gene expressions, immune cell infiltrations, and activation of immune pathways, the higher degree of immune cell infiltrations enables it to achieve better results in immunotherapy, proving that our definition of the low-risk group as the high-immunity group in terms of immunotherapeutic effects is correct. Then, TMB values of response and non-response samples were determined, which did not reveal significant differences in TMB values (**Figure 10B**). The GRGPI established in this paper is superior to TMB in predicting immunotherapeutic effects. To validate the effects of immunotherapy in IMvigor210, differences in GRGPIs between response and non-response samples were investigated and found to be insignificant (**Figure 10C**). Differences between the two groups of TMB values were analyzed, and the response group was found to have higher TMB values (**Figure 10D**).

Therefore, the proportion of response and non-response samples in the three subgroups was identified after combining GRGPI and TMB (**Figure 10E**). The HTMB+HGRGPI group had the most significant proportion of responding models, followed by HTMB+LGRGPI& LTMB+HGRGPI, and LTMB+LGRGPI, suggesting that immunotherapy had better effects in the HTMB+HGRGPI group. To determine the prognostic performance of the established three subgroup models, we compared the AUC values of the three predictive models of GRGPI, TMB, and GRGPI combined with TMB (**Figure 10F**), which were 0.534, 0.647, and 0.646. The TMB and GRGPI combined with TMB exhibited better predictive performance. Finally, the prognostic value of the predictive model in melanoma was assessed using the GSE78220 cohort for external validation. Differences in GRGPIs between response and non-response groups were insignificant (**Supplementary Figure 5**).

### 3.14 Drug sensitivity

Although ICI therapy has shown great promise for the treatment of HNSCC, given its high costs and limited therapeutic effects (326/457 showed no responses to ICI therapy in this study), chemotherapy is a clinically meaningful treatment. However, HNSCC is associated with significant resistance to chemotherapeutic drugs during clinical treatment. To assess the application effects in the clinical chemotherapy process of the established model, IC50 was used to express the sensitivity of the high-risk and low-risk groups to several common chemotherapeutic drugs. Cisplatin, paclitaxel, and docetaxel were recommended by the Chinese Society of Clinical Oncology (CSCO) Guidelines of 2021 as first-line therapeutic drugs for HNSCC. Therefore, IC50 values in a high-risk group and low-risk group of the three drugs were calculated (**Figures 10G–I**). Patients in the high-risk group were more sensitive to cisplatin (p=1.4e-05) and docetaxel (p=5.5e-12). In contrast, those in the low-risk group were more sensitive to paclitaxel (p=9.9e-01), implying that the established model indicates chemotherapeutic sensitivity.

### 3.15 RT-qPCR analysis

**Figure 11:**
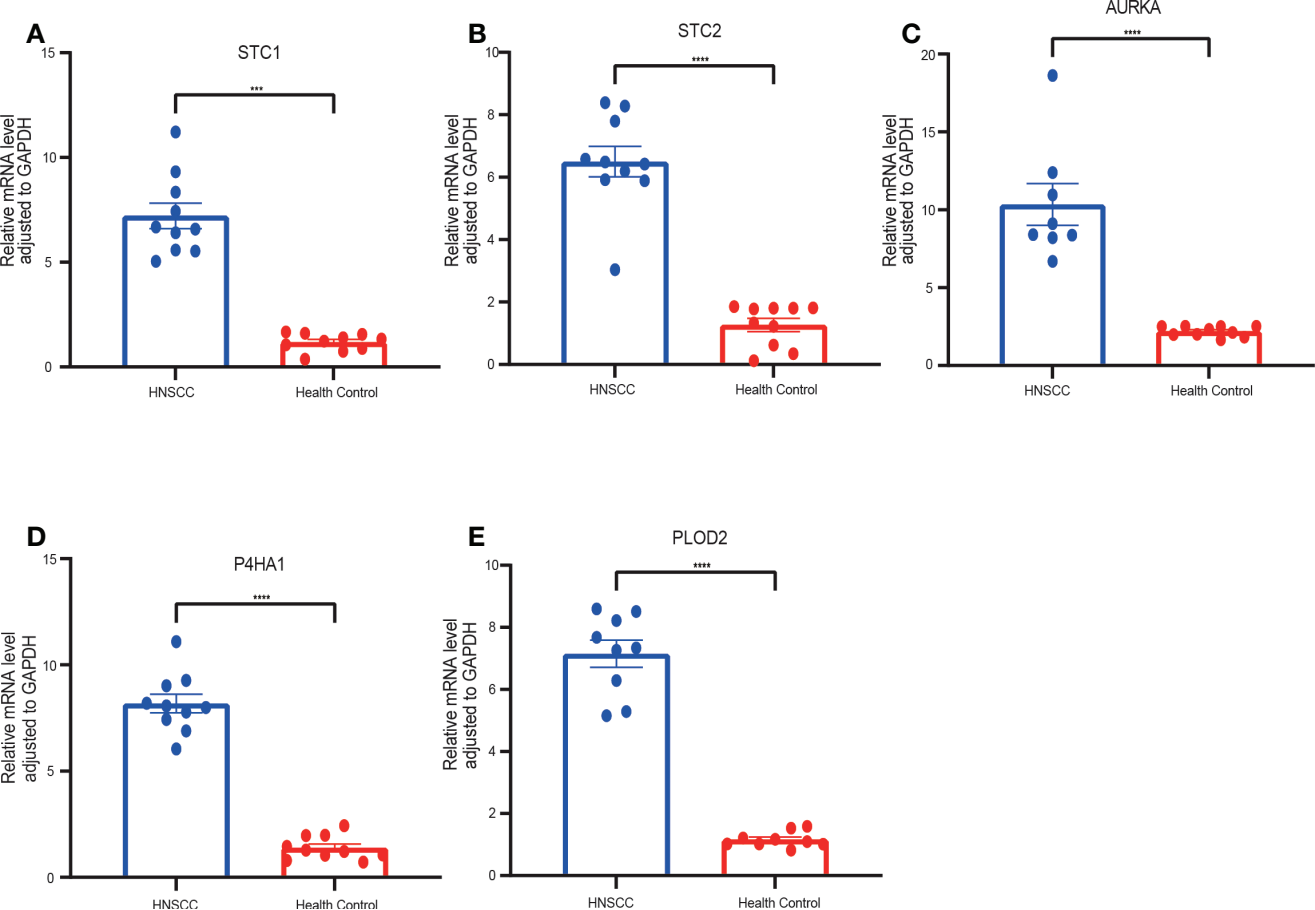
RT-qPCR analyses of five hub genes between HNSCC and Healthy control tissues. Relative mRNA expressions of **(A)** STC1, **(B)** STC2, **(C)** AURKA, **(D)** P4HA1, **(E)** PLOD2. (***p < 0.001,****p < 0.0001).

To verify the accuracy of GRGPI in HNSCC patients, we collected HNSCC tissues and adjacent non-tumorous tissues from 10 HNSCC patients. RT-qPCR was implemented to analyze the expressions of five prognostic glycolysis-related genes in the GRGPI. We found that compared with adjacent non-tumorous tissues, the terms of five genes were significantly up-regulated in HNSCC tissues (**Figures 11A–E**).

## 4 Discussion

Conversion of the primary energy source from oxidative phosphorylation (OXPHOS) to aerobic glycolysis is an emerging hallmark of cancer cells (22). Although the amount of ATP produced by glycolysis is low, several advantages inherent to aerobic glycolysis can explain this metabolic switch in cancer cells. Glycolysis produces ATP 100 times faster than OXPHOS (23), which can provide sufficient energy for cell survival. Second, glycolytic intermediates can be transferred to various biosynthetic pathways to provide materials for the synthesis of biomolecules and organelles (24, 25). In addition, glutathione is key in protecting cancer cells from oxidative damage and antitumor drugs. In contrast, intermediates accumulated by cancer cells during glycolysis promote the pentose phosphate pathway and can ensure their growth in an environment with reduced glutathione levels (26, 27). Finally, the formation of an acidic microenvironment associated with lactate accumulation due to increased glycolysis provides a tissue environment for tumor recurren tumor metastasis (28). These factors increase the dependence of tumor cells on glycolysis and provide a biochemical basis for the preferential killing of cancer cells by using glycolysis as a therapeutic target, possibly resulting in improved therapeutic efficacies (29).

Studies are evaluating the molecular mechanisms of glycolysis in tumorigenesis, proliferation, and invasion. For instance, PLOD2 induces epithelial-mesenchymal transition (EMT) *via* the PI3K/AKT signaling pathway. It is involved in regulating outer stromal collagen and tumor metastasis through EMT, TGF-β, and hypoxic signaling. PLOD2 levels are significantly associated with advanced cancer staging. The presence of regional STC1 uncouples the oxidative phosphorylation process by increasing the expressions of mitochondrial UCP2, which is a valuable biomarker for the diagnosis of malignant glioma for the assessment of postoperative prognosis. Elevated STC2 levels selectiveprotectcts HeLa cells from endoplasmic reticulum stress-induced cell death and are also associated with larger tumor formation, tumor invasion, lymph node metastasis, and poor prognostic outcomes. P4HA1 is a hypoxia-responsive gene that plays a key role in regulating collagen biosynthesis (30). HPV infections promote HNSCC by suppressing P4HA1. AURKA-mediated phosphorylation can regulate the function of AURKA-discovered substrates, some of which are filamentous regulators, tumor suppressors, or factors in cancer. There are already several small molecules targeting AURKA that have been tested in AURKA (AKI) preclinical studies (31).

Given the importance of glycolysis in HNSCC, it can be hypothesized that glycolysis-related genes are potential prognostic factors for HNSCC. In addition, computed multigene prognostic markers outperformed single biomarkers in predicting overall survival. We analyzed the mRNA expression profiles of 49 glycolysis-related genes in the TCGA head and neck squamous cell carcinoma cohort. Five genes associated with glycolysis were selected as candidate prognostic factors for HNSCC. These genes are potential molecular predictive biomarkers and may help inform individualized treatments based on patient risk. We combined the established risk scores and multiple clinical parameters to construct column line plots for predicting the 1-year, 3-year, and 5-year OS in HNSCC patients. Calibration plots based on the TCGA database showed that the expected and observed values were very close, indicating the excellent predictive performance of column line plots. The predictive efficacy was equally good when examined in the validation set. Thus, our new prognostic column line plot may be better than the original clinical factors for predicting survival status for HNSCC patients and informing specific individualized treatment.

Analysis of the new risk scoring model (GRGPI) revealed higher immune cell infiltration scores in the low-risk group. Host immunosuppression is an integral factor in HNSCC carcinogenesis (32). The immune microenvironment is characterized by the presence of infiltrating immune cells (33). We compared immune cell infiltrations between the high-risk and low-risk groups of HNSCC. We found that resting CD4 memory cells, M0-phase macrophages, M2-phase macrophages, and activated mast cells were highly infiltrated in the high-risk group. In contrast, Tregs and other cells were more in the low-risk group. Acquired immune-related cell infiltrations were lower in the high-risk group compared to the low-risk group, suggesting that the higher risk score may be associated with immunosuppression. CD8 T cells directly targeting tumor cells were more stable in the low-risk group. However, CD4 T cells in the tumor microenvironment were unstable for a broad subpopulation with potentially different functions (34). CTLA-4, which is downregulated in the low-immune group, is the first negative regulator of T cell activation identified in the context of antitumor immunity, and its blockade using monoclonal antibodies triggers tumor regression with durable antitumor immunity in preclinical models. LAG-3 acts synergistically with other checkpoint molecules to promote T cell dysfunction. However, the molecular mechanisms and pathways associated with LAG-3 signaling have not been fully established (35). In this regard, the conserved KIEELE motif in the cytoplasmic structural domain was indispensable for LAG-3 downstream signaling and inhibition of CD4 T cell activation. MHC-II/LAG-3 triggers the activation of ITAM signaling in DCs, thereby promoting a tolerance profile (36). Thus, MHC-II/LAG-3 interactions function as a bidirectional inhibitory pathway.

Through immune pathway analyses, cytokines, TGF-β family, and TGF-β family receptors were activated in the high-risk group of the TCGA dataset. The postulate that overproduction of TGF-β promotes tumor progression was verified. While the TGF-β-related pathway plays an important role in inhibiting the proliferation of immunoreactive cells and stimulating the expressions of the extracellular matrix, activation of the TGF-β-related pathway in the high-risk group may be one of the reasons for the immunosuppression and lower stromal scores. Immune cell dysfunctions within the HNSCC-TME promote immunosuppression and may thus be associated with tumor survival and progression outcomes. Therefore, it also requires therapeutic interventions (37, 38). We found that the density of CD8 T cells, resting dendritic cells, follicular helper T cells, Treg cells, and high immune scores correlated with patient prognosis, consistent with findings from previous studies (20, 39). This underscores the fact that preexisting immune responses have antitumor effects and positively influence immunotherapeutic responses. Several seminal clinical and genomic studies have reported that HNSCC has a high degree of immune cell infiltrations. However, less than 20% of HNSCC patients respond to immunotherapy, implying that even the resistant phenotype in the tumor is not an absolute predictor of immunotherapeutic responses (40, 41). Molecular analyses of HNSCC have identified a range of cytokines, chemokines, and other TME components that determine the ability of the host to mount anti-tumor immune responses. During tumorigenesis, these molecular changes may interfere with intercellular communication between infiltrating immune cells, disrupting the balance between immune tolerance and cellular activity (42).

Higher CYT scores were associated with higher expressions of inhibitory ligands by tumor cells that predispose to immune evasion. Patients with high CYT scores showed better efficacies regarding checkpoint inhibitors such as PD-L1 than those with low CYT scores. Based on previous risk groupings, we found that the low-risk group had higher CYT scores (43), suggesting that immune cells in the low-risk group had stronger cytolytic activities and antitumor immune responses may have better prognostic outcomes. Drug sensitivity assays revealed that patients in the high-risk group were more sensitive to cisplatin and docetaxel. In contrast, patients in the low-risk group were more sensitive to paclitaxel, gefitinib, and erlotinib, suggesting that this model can be used as a potential predictor of chemotherapeutic sensitivity for screening sensitive drugs. Tumor cell chemotherapy drug sensitivity testing can provide valuable information to physicians to support their treatment decisions and provide a powerful tool for physicians and patients in their battle against cancer.

Overall, according to survival analysis, functional analysis, ICI therapy, drug sensitivity, and RT-qPCR analysis, the signature was a valuable indicator for predicting survival outcomes among HNSCC patients. But our study still has some limitations. First, it was carried out based on the TCGA database, which lacked specific data on surgery, chemotherapy, and tumor size. Besides, some patients have undergone immune or targeted therapy, which may impact the prognosis analysis. Second, a very high proportion of patients with tumors located in the oral cavity in the model could make it difficult to generalize the results of head and neck cancer. Third, the number of patients we collected was too small to validate the performance of our prognostic model.

## 5 Conclusion

In conclusion, a new HNSCC prognostic signature based on five glycolysis-related genes was constructed in the TCGA cohort and validated in the GEO database. The signature shows excellent performance in predicting survival outcomes among HNSCC patients, reveals the relationship between glycolysis-related genes and tumor immunity in HNSCC and provides guidance to clinical treatment decisions.

## Data availability statement

The original contributions presented in the study are included in the article/**Supplementary Material**. Further inquiries can be directed to the corresponding authors.

## Ethics statement

The studies involving human participants were reviewed and approved by Cancer Hospital Affiliated to Shanxi Medical University. The patients/participants provided their written informed consent to participate in this study.

## Author contributions

Conceived and designed the study: FH, H-ZW, M-JC, P-FH, X-TY, FLi. Collected and analyzed the data: Y-TH and M-JC. Performed the experiments and verificate experiments: H-ZW and FH. Wrote the initial draft: L-ZL, SL. The corresponding authors are responsible for ensuring that the descriptions are accurate and agreed by all authors. All authors contributed to the article and approved the submitted version.

## References

[1] Gillison ML Chaturvedi AK Anderson WF Fakhry C. Epidemiology of human papillomavirus-positive head and neck squamous cell carcinoma. J Clin Oncol (2015) 33(29):3235–42. doi: 10.1200/JCO.2015.61.6995 PMC4979086 26351338

[2] Chow LQM. Head and neck cancer. New Engl J Med (2020) 382(1):60–72. doi: 10.1056/NEJMra1715715 31893516

[3] Vaupel P Schmidberger H Mayer A. The warburg effect: Essential part of metabolic reprogramming and central contributor to cancer progression. Int J Radiat Biol (2019) 95(7):912–9. doi: 10.1080/09553002.2019.1589653 30822194

[4] Lu J Tan M Cai Q. The warburg effect in tumor progression: Mitochondrial oxidative metabolism as an anti-metastasis mechanism. Cancer Lett (2015) 356(2 Pt A):156–64. doi: 10.1016/j.canlet.2014.04.001 PMC4195816 24732809

[5] Huebbers CU Adam AC Preuss SF Schiffer T Schilder S Guntinas-Lichius O. High glucose uptake unexpectedly is accompanied by high levels of the mitochondrial s-F1-Atpase subunit in head and neck squamous cell carcinoma. Oncotarget (2015) 6(34):36172–84. doi: 10.18632/oncotarget.5459 PMC4742169 26452026

[6] Chen L He X Yi S Liu G Liu Y Ling Y. Six glycolysis-related genes as prognostic risk markers can predict the prognosis of patients with head and neck squamous cell carcinoma. BioMed Res Int (2021) 2021:8824195. doi: 10.1155/2021/8824195 33628816 PMC7889344

[7] Yang J-G Wang W-M Xia H-F Yu Z-L Li H-M Ren J-G. Lymphotoxin-A promotes tumor angiogenesis in hnscc by modulating glycolysis in a Pfkfb3-dependent manner. Int J Cancer (2019) 145(5):1358–70. doi: 10.1002/ijc.32221 30785217

[8] Ganapathy-Kanniappan S Geschwind JF. Tumor glycolysis as a target for cancer therapy: Progress and prospects. Mol Cancer (2013) 12:152. doi: 10.1186/1476-4598-12-152 24298908 PMC4223729

[9] Akram M. Mini-review on glycolysis and cancer. J Cancer Educ (2013) 28(3):454–7. doi: 10.1007/s13187-013-0486-9 23728993

[10] Kumar D. Regulation of glycolysis in head and neck squamous cell carcinoma. Postdoc J (2017) 5(1):14–28. doi: 10.14304/surya.jpr.v5n1.4 28191478 PMC5300767

[11] Vivanco I Sawyers CL. The phosphatidylinositol 3-kinase akt pathway in human cancer. Nat Rev Cancer (2002) 2(7):489–501. doi: 10.1038/nrc839 12094235

[12] Elstrom RL Bauer DE Buzzai M Karnauskas R Harris MH Plas DR. Akt stimulates aerobic glycolysis in cancer cells. Cancer Res (2004) 64(11):3892–9. doi: 10.1158/0008-5472.Can-03-2904 15172999

[13] Schreiber RD Old LJ Smyth MJ. Cancer immunoediting: Integrating immunity's roles in cancer suppression and promotion. Science (2011) 331(6024):1565–70. doi: 10.1126/science.1203486 21436444

[14] Dunn GP Old LJ Schreiber RD. The immunobiology of cancer immunosurveillance and immunoediting. Immunity (2004) 21(2):137–48. doi: 10.1016/j.immuni.2004.07.017 15308095

[15] Jie H-B Srivastava RM Argiris A Bauman JE Kane LP Ferris RL. Increased pd-1 and Tim-3 tils during cetuximab therapy inversely correlate with response in head and neck cancer patients. Cancer Immunol Res (2017) 5(5):408–16. doi: 10.1158/2326-6066 PMC5497750 28408386

[16] Bell RB Leidner RS Crittenden MR Curti BD Feng Z Montler R. OX40 signaling in head and neck squamous cell carcinoma: Overcoming immunosuppression in the tumor microenvironment. Oral Oncol (2016) 52:1ߝ10. doi: 10.1016/j.oraloncology.2015.11.009 26614363

[17] Caponio VCA Troiano G Adipietro I Zhurakivska K Arena C Mangieri D. Computational analysis of Tp53 mutational landscape unveils key prognostic signatures and distinct pathobiological pathways in head and neck squamous cell cancer. Br J Cancer (2020) 123(8):1302–14. doi: 10.1038/s41416-020-0984-6 PMC7553957 32684626

[18] Ahmadzadeh M Rosenberg SA. Tgf-beta 1 attenuates the acquisition and expression of effector function by tumor antigen-specific human memory Cd8 T cells. J Immunol (Baltimore Md 1950) (2005) 174(9):5215–23. doi: 10.4049/jimmunol.174.9.5215 PMC2562293 15843517

[19] Danaher P Warren S Lu R Samayoa J Sullivan A Pekker I. Pan-cancer adaptive immune resistance as defined by the tumor inflammation signature (Tis): Results from the cancer genome atlas (Tcga). J For Immunotherapy Cancer (2018) 6(1):63. doi: 10.1186/s40425-018-0367-1 PMC6013904 29929551

[20] Rooney MS Shukla SA Wu CJ Getz G Hacohen N. Molecular and genetic properties of tumors associated with local immune cytolytic activity. Cell (2015) 160(1-2):48–61. doi: 10.1016/j.cell.2014.12.033 25594174 PMC4856474

[21] Jiang P Gu S Pan D Fu J Sahu A Hu X. Signatures of T cell dysfunction and exclusion predict cancer immunotherapy response. Nat Med (2018) 24(10):1550–8. doi: 10.1038/s41591-018-0136-1 PMC6487502 30127393

[22] Hanahan D Weinberg RA. Hallmarks of cancer: The next generation. Cell (2011) 144(5):646–74. doi: 10.1016/j.cell.2011.02.013 21376230

[23] Locasale JW Cantley LC. Altered metabolism in cancer. BMC Biol (2010) 8:88. doi: 10.1186/1741-7007-8-88 20598111 PMC2892450

[24] Deberardinis RJ Sayed N Ditsworth D Thompson CB. Brick by brick: Metabolism and tumor cell growth. Curr Opin In Genet Dev (2008) 18(1):54–61. doi: 10.1016/j.gde.2008.02.003 18387799 PMC2476215

[25] Whitaker-Menezes D Martinez-Outschoorn UE Lin Z Ertel A Flomenberg N Witkiewicz AK. Evidence for a stromal-epithelial "Lactate shuttle" in human tumors: Mct4 is a marker of oxidative stress in cancer-associated fibroblasts. Cell Cycle (Georgetown Tex) (2011) 10(11):1772–83. doi: 10.4161/cc.10.11.15659 PMC3142461 21558814

[26] Pacini N Borziani F. Cancer stem cell theory and the warburg effect, two sides of the same coin? Int J Mol Sci (2014) 15(5):8893–930. doi: 10.3390/ijms15058893 PMC4057766 24857919

[27] Traverso N Ricciarelli R Nitti M Marengo B Furfaro AL Pronzato MA. Role of glutathione in cancer progression and chemoresistance. Oxid Med Cell Longevity (2013) 2013:972913. doi: 10.1155/2013/972913 PMC3673338 23766865

[28] Pelicano H Martin DS Xu RH Huang P. Glycolysis inhibition for anticancer treatment. Oncogene (2006) 25(34):4633–46. doi: 10.1038/sj.onc.1209597 16892078

[29] Sacco AG Cohen EE. Current treatment options for recurrent or metastatic head and neck squamous cell carcinoma. J Clin Oncol Off J Am Soc Clin Oncol (2015) 33(29):3305–13. 10.1200/JCO.2015.62.0963 26351341

[30] Li Q Shen Z Wu Z Shen Y Deng H Zhou C. High P4ha1 expression is an independent prognostic factor for poor overall survival and recurrent-free survival in head and neck squamous cell carcinoma. J Clin Lab Anal (2020) 34(3):e23107. doi: 10.1002/jcla.23107 31782831 PMC7083458

[31] Du R Huang C Liu K Li X Dong Z. Targeting aurka in cancer: Molecular mechanisms and opportunities for cancer therapy. Mol Cancer (2021) 20(1):15. doi: 10.1186/s12943-020-01305-3 33451333 PMC7809767

[32] Tsukamoto H Fujieda K Miyashita A Fukushima S Ikeda T Kubo Y. Combined blockade of Il6 and pd-1/Pd-L1 signaling abrogates mutual regulation of their immunosuppressive effects in the tumor microenvironment. Cancer Res (2018) 78(17):5011–22. doi: 10.1158/0008-5472.CAN-18-0118 29967259

[33] Maggioni D Pignataro L Garavello W. T-Helper and T-regulatory cells modulation in head and neck squamous cell carcinoma. Oncoimmunology (2017) 6(7):e1325066. doi: 10.1080/2162402X.2017.1325066 28811959 PMC5543829

[34] de Ruiter EJ Ooft ML Devriese LA Willems SM. The prognostic role of tumor infiltrating T-lymphocytes in squamous cell carcinoma of the head and neck: A systematic review and meta-analysis. Oncoimmunology (2017) 6(11):e1356148. doi: 10.1080/2162402X.2017.1356148 29147608 PMC5674970

[35] Workman CJ Dugger KJ Vignali DAA. Cutting edge: Molecular analysis of the negative regulatory function of lymphocyte activation gene-3. J Immunol (Baltimore Md 1950) (2002) 169(10):5392–5. doi: 10.4049/jimmunol.169.10.5392 12421911

[36] Liang B Workman C Lee J Chew C Dale BM Colonna L. Regulatory T cells inhibit dendritic cells by lymphocyte activation gene-3 engagement of mhc class ii. J Immunol (Baltimore Md: 1950) (2008) 180(9):5916–26. doi: 10.4049/jimmunol.180.9.5916 18424711

[37] Czystowska M Gooding W Szczepanski MJ Lopez-Abaitero A Ferris RL Johnson JT. The immune signature of Cd8(+)Ccr7(+) T cells in the peripheral circulation associates with disease recurrence in patients with hnscc. Clin Cancer Res an Off J Am Assoc For Cancer Res (2013) 19(4):889–99. doi: 10.1158/1078-0432.CCR-12-2191 PMC3708459 23363813

[38] Davis RJ Van Waes C Allen CT. Overcoming barriers to effective immunotherapy: Mdscs, tams, and tregs as mediators of the immunosuppressive microenvironment in head and neck cancer. Oral Oncol (2016) 58:59–70. doi: 10.1016/j.oraloncology.2016.05.002 27215705 PMC4912416

[39] Mandal R Şenbabaoğlu Y Desrichard A Havel JJ Dalin MG Riaz N. The head and neck cancer immune landscape and its immunotherapeutic implications. JCI Insight (2016) 1(17):e89829. doi: 10.1172/jci 27777979 PMC5070962

[40] Seiwert TY Burtness B Mehra R Weiss J Berger R Eder JP. Safety and clinical activity of pembrolizumab for treatment of recurrent or metastatic squamous cell carcinoma of the head and neck (Keynote-012): An open-label, multicentre, phase 1b trial. Lancet Oncol (2016) 17(7):956–65. doi: 10.1016/S1470-2045(16)30066-3 27247226

[41] Yarchoan M Hopkins A Jaffee EM. Tumor mutational burden and response rate to pd-1 inhibition. New Engl J Med (2017) 377(25):2500–1. doi: 10.1056/NEJMc1713444 PMC6549688 29262275

[42] Şenbabaoğlu Y Gejman RS Winer AG Liu M Van Allen EM de Velasco G. Tumor immune microenvironment characterization in clear cell renal cell carcinoma identifies prognostic and immunotherapeutically relevant messenger rna signatures. Genome Biol (2016) 17(1):231. doi: 10.1186/s13059-016-1092-z 27855702 PMC5114739

[43] Gao Z Tao Y Lai Y Wang Q Li Z Peng S. Immune cytolytic activity as an indicator of immune checkpoint inhibitors treatment for prostate cancer. Front In Bioengineering Biotechnol (2020) 8:930. doi: 10.3389/fbioe.2020.00930 PMC7423880 32850758

