# Supplementary figures and images for: Development and validation of a GRGPI model for predicting the prognostic and treatment outcomes in head and neck squamous cell carcinoma
Source: Front Oncol. 2023 Jan 12;12:972215. doi: 10.3389/fonc.2022.972215 (PMC9877611; doi:10.3389/fonc.2022.972215)

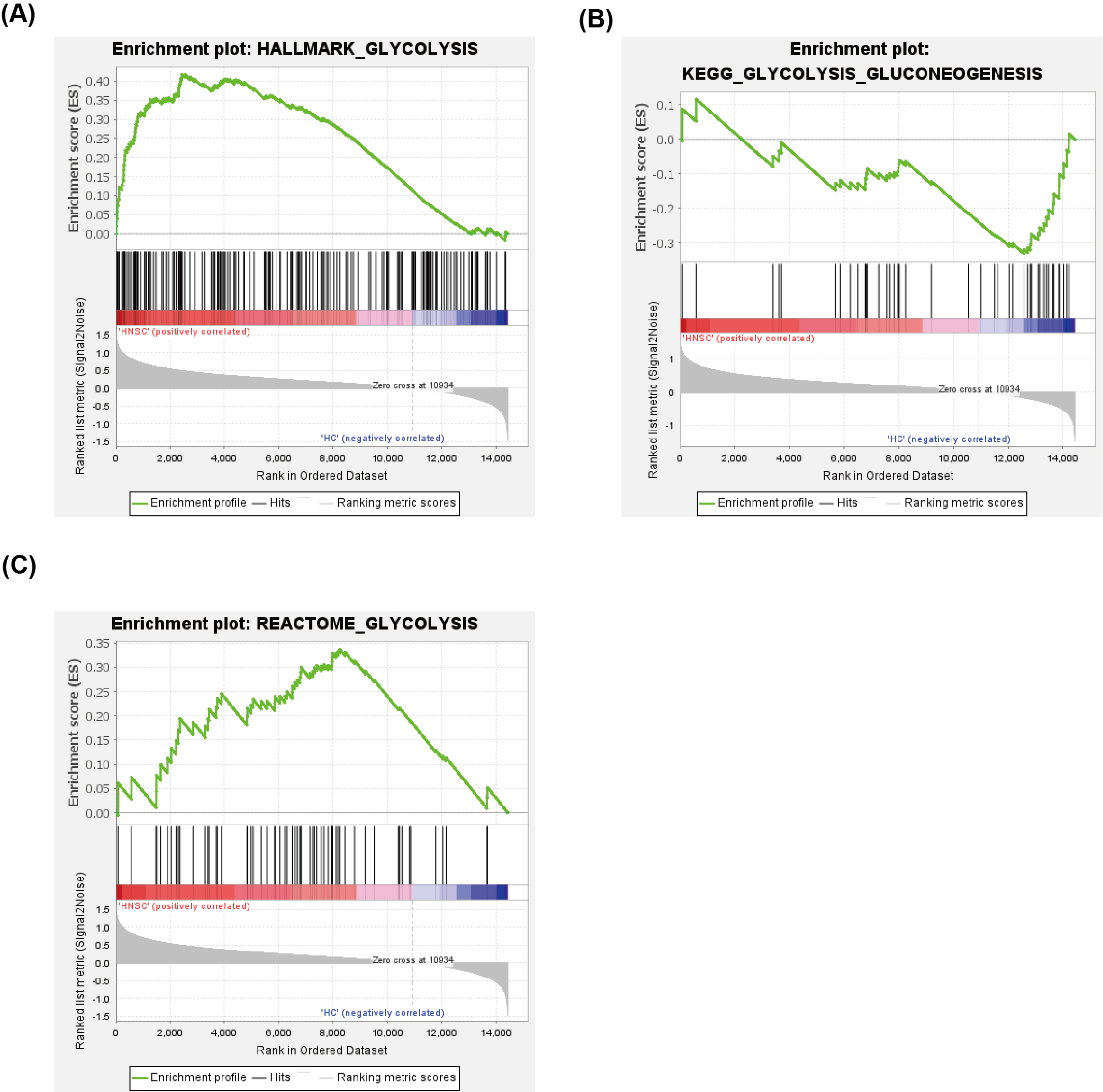

Supplement: Supplementary file 1 [file Image_1.jpeg]

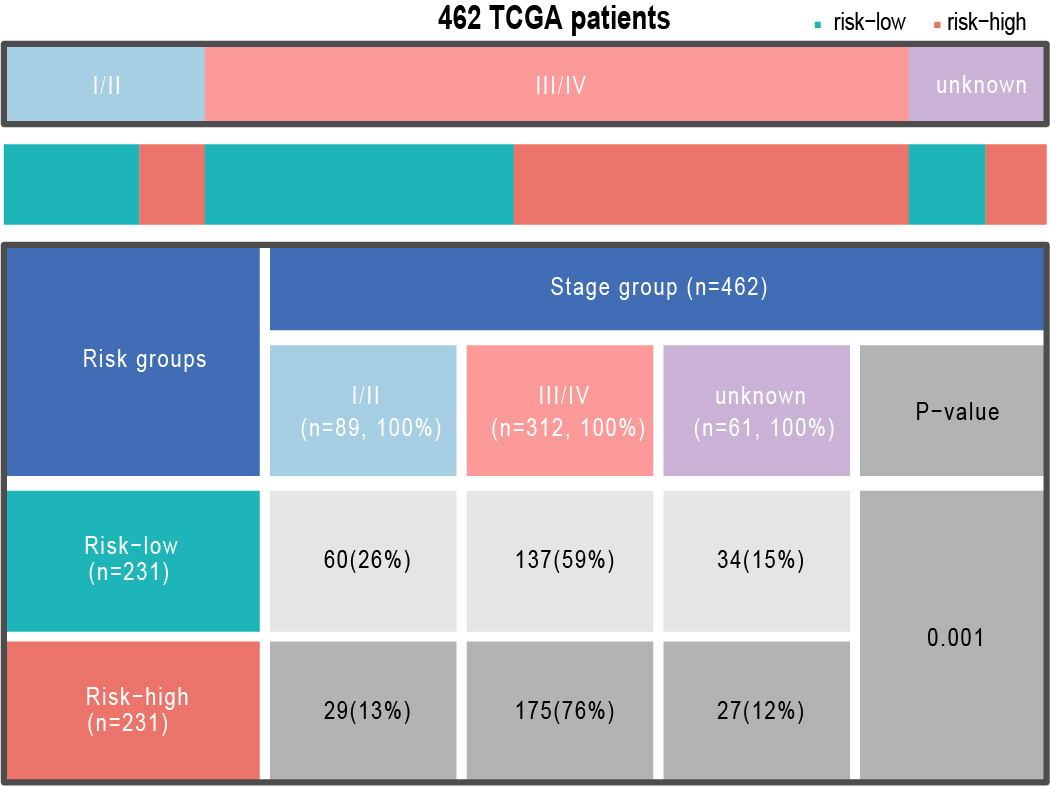

Supplement: Supplementary file 2 [file Image_2.jpeg]

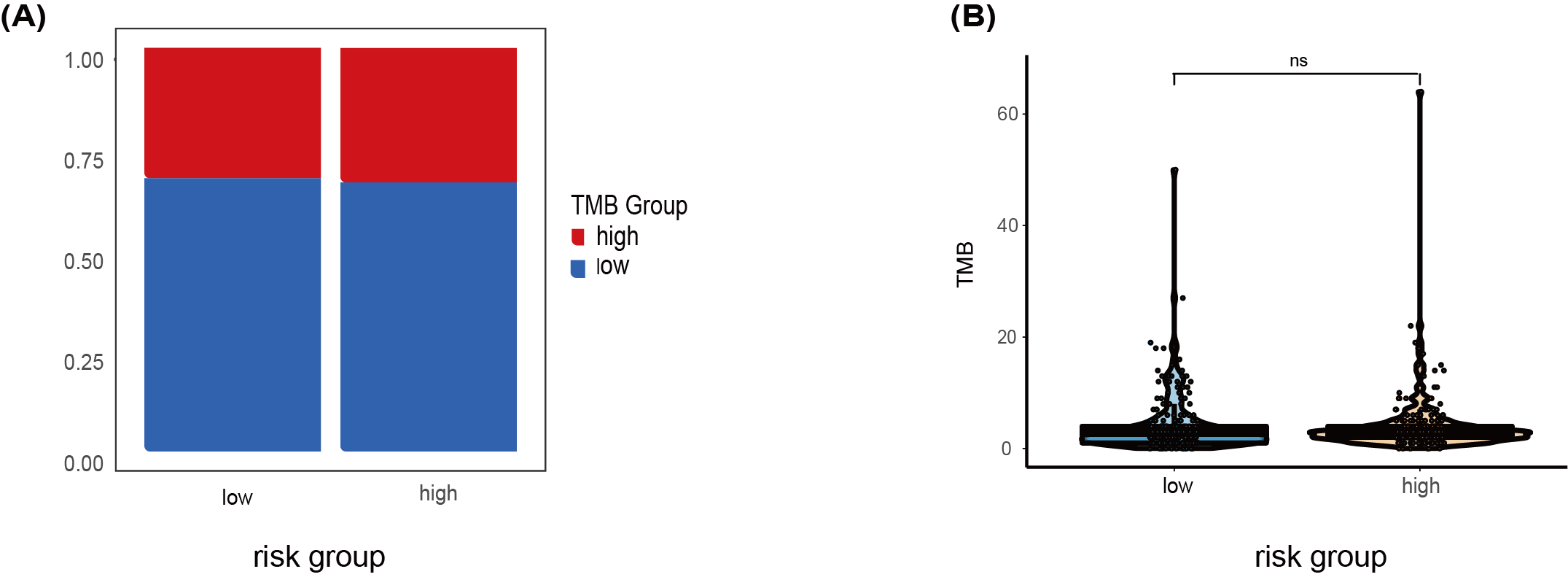

Supplement: Supplementary file 3 [file Image_3.jpeg]

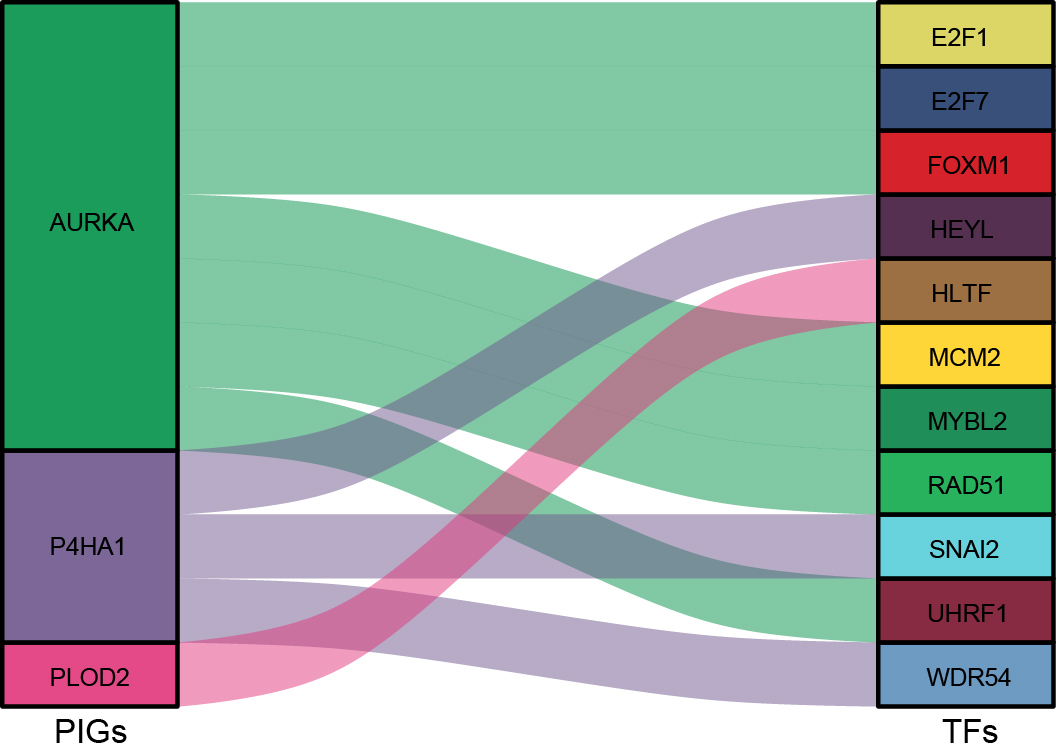

Supplement: Supplementary file 4 [file Image_4.jpeg]

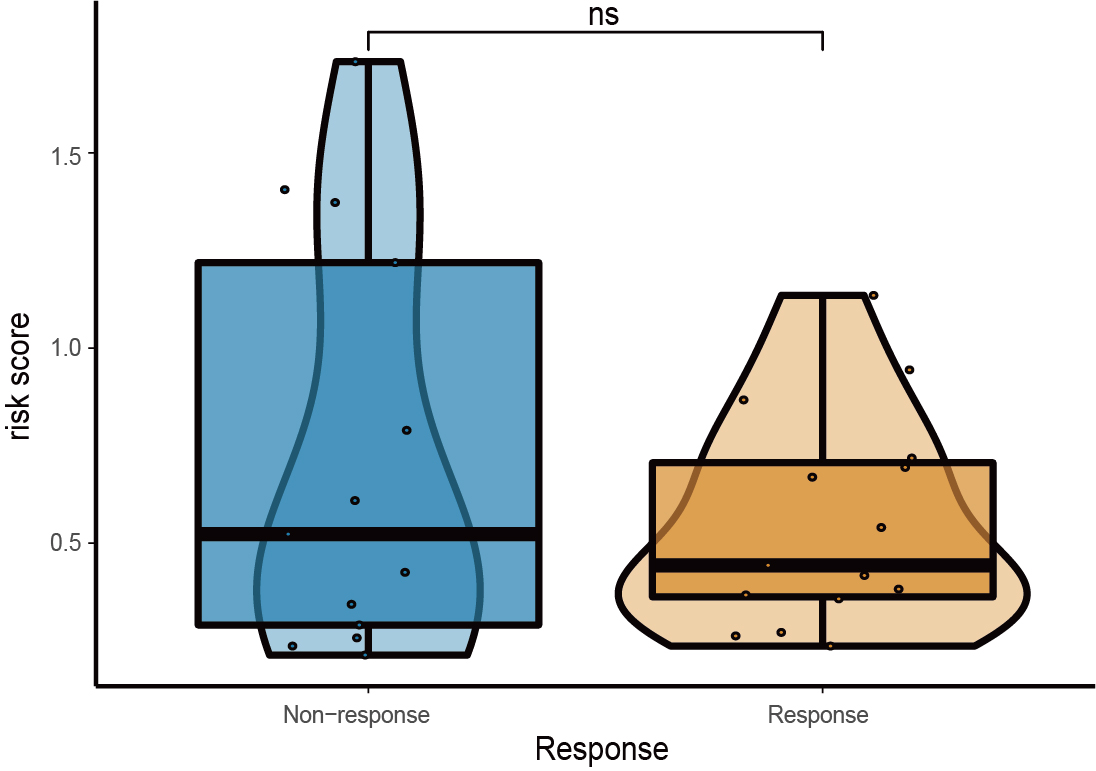

Supplement: Supplementary file 5 [file Image_5.jpeg]
